# (*E*)-2-[4-(Di­ethyl­amino)­styr­yl]-1-methyl­quinolin-1-ium 4-chloro­benzene­sulfonate monohydrate

**DOI:** 10.1107/S1600536814004577

**Published:** 2014-03-05

**Authors:** Suchada Chantrapromma, Narissara Kaewmanee, Nawong Boonnak, Ching Kheng Quah, Hoong-Kun Fun

**Affiliations:** a Department of Chemistry, Faculty of Science, Prince of Songkla University, Hat-Yai, Songkhla 90112, Thailand; b Faculty of Traditional Thai Medicine, Prince of Songkla University, Hat-Yai, Songkhla 90112, Thailand; c X-ray Crystallography Unit, School of Physics, Universiti Sains Malaysia, 11800 USM, Penang, Malaysia

## Abstract

The asymmetric unit of the title hydrated salt, C_22_H_25_N_2_^+^·C_6_H_4_ClO_3_S^−^·H_2_O, comprises two 2-[4-(di­ethyl­amino)­styr­yl]-1-methyl­quinolin-1-ium cations, two 4-chloro­benzene­sul­fon­ate anions and two solvent water mol­ecules. One ethyl group of both cations displays disorder over two positions in a 0.659 (2):0.341 (2) ratio in one mol­ecule and in a 0.501 (2):0.499 (2) ratio in the other. The sulfonate group of one anion is also disordered over two positions in a 0.893 (7):0.107 (7) ratio. The dihedral angle between the mean plane of the quinolinium ring system and that of benzene ring is 10.57 (18)° in one cation and 14.4 (2)° in the other. In the crystal, cations, anions and water mol­ecules are linked into chains along the [010] direction by O—H⋯O_sulfonate_ hydrogen bonds, together with weak C—H⋯O_sulfonate_ and C—H⋯Cl inter­actions. The cations are stacked by π–π inter­actions, with centroid–centroid distances in the range 3.675 (2)–4.162 (3) Å.

## Related literature   

For standard bond lengths, see: Allen *et al.* (1987 ▶). For background to and applications of quarternary ammonium compounds, see: Barchéchath *et al.* (2005 ▶); Chanawanno *et al.* (2010*a* ▶,*b* ▶); Bolden *et al.* (2013 ▶). For related structures, see: Chantrapromma *et al.* (2012 ▶); Fun, Kaewmanee *et al.* (2011 ▶, 2013 ▶); Kaewmanee *et al.* (2010 ▶). For the stability of the temperature controller used in the data collection, see: Cosier & Glazer (1986 ▶).
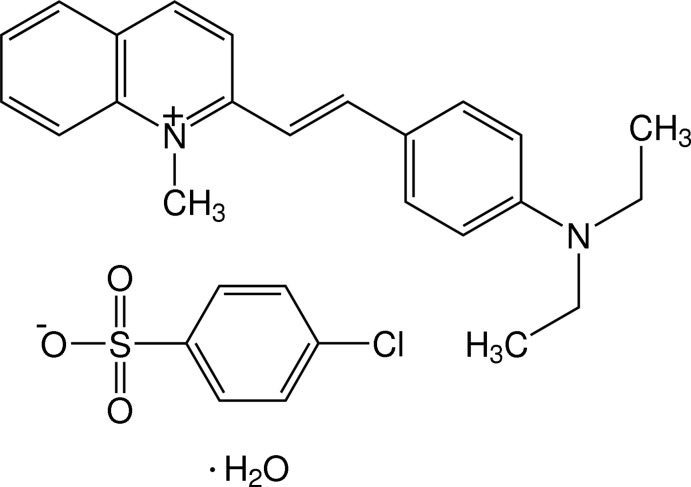


## Experimental   

### 

#### Crystal data  

C_22_H_25_N_2_^+^·C_6_H_4_ClO_3_S^−^·H_2_O
*M* *_r_* = 527.07
Monoclinic, 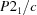

*a* = 25.814 (4) Å
*b* = 10.5563 (16) Å
*c* = 20.333 (3) Å
β = 110.883 (2)°
*V* = 5176.8 (14) Å^3^
*Z* = 8
Mo *K*α radiation
μ = 0.27 mm^−1^
*T* = 100 K
0.31 × 0.19 × 0.15 mm

#### Data collection  

Bruker SMART APEXII DUO CCD area-detector diffractometer
Absorption correction: multi-scan (*SADABS*; Bruker, 2009 ▶) *T*_min_ = 0.923, *T*_max_ = 0.961
28821 measured reflections
10657 independent reflections
6269 reflections with *I* > 2σ(*I*)
*R*_int_ = 0.064

#### Refinement  

*R*[*F* ^2^ > 2σ(*F* ^2^)] = 0.076
*wR*(*F* ^2^) = 0.201
*S* = 1.04
10657 reflections
708 parameters
H-atom parameters constrained
Δρ_max_ = 0.61 e Å^−3^
Δρ_min_ = −0.43 e Å^−3^

### 

Data collection: *APEX2* (Bruker, 2009 ▶); cell refinement: *APEX2*; data reduction: *SAINT* (Bruker, 2009 ▶); program(s) used to solve structure: *SHELXTL* (Sheldrick, 2008 ▶); program(s) used to refine structure: *SHELXTL*; molecular graphics: *SHELXTL*; software used to prepare material for publication: *SHELXTL*, *PLATON* (Spek, 2009 ▶), *Mercury* (Macrae *et al.*, 2008 ▶) and *publCIF* (Westrip, 2010 ▶).

## Supplementary Material

Crystal structure: contains datablock(s) global, I. DOI: 10.1107/S1600536814004577/sj5390sup1.cif

Structure factors: contains datablock(s) I. DOI: 10.1107/S1600536814004577/sj5390Isup2.hkl

Supporting information file. DOI: 10.1107/S1600536814004577/sj5390Isup3.cml

CCDC reference: 988937

Additional supporting information:  crystallographic information; 3D view; checkCIF report

## Figures and Tables

**Table 1:** Hydrogen-bond geometry (Å, °)

*D*—H⋯*A*	*D*—H	H⋯*A*	*D*⋯*A*	*D*—H⋯*A*
O1*WB*—H1*WB*⋯O1*B* ^i^	0.85	2.36	2.815 (7)	114
O1*WB*—H2*WB*⋯O2*B* ^ii^	0.83	2.12	2.953 (7)	177
O1*WA*—H1*WA*⋯O2*A* ^iii^	0.84	2.07	2.891 (5)	166
O1*WA*—H2*WA*⋯O1*A*	0.76	2.10	2.844 (4)	169
C8*A*—H8*AA*⋯O3*A* ^iv^	0.93	2.54	3.146 (5)	123
C2*B*—H2*BA*⋯O3*B* ^v^	0.93	2.57	3.314 (7)	137
C11*B*—H11*B*⋯O1*B* ^vi^	0.93	2.41	3.237 (6)	148
C18*Y*—H18*E*⋯Cl1*A* ^vii^	0.97	2.72	3.673 (19)	169
C19*B*—H19*D*⋯Cl1*B* ^viii^	0.96	2.73	3.531 (14)	142
C22*B*—H22*D*⋯O2*B* ^viii^	0.96	2.55	3.259 (7)	131
C25*A*—H25*A*⋯O3*A* ^ii^	0.93	2.56	3.359 (5)	144

Symmetry codes: (i) 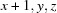; (ii) 
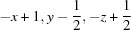
; (iii) 
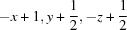
; (iv) 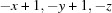; (v) 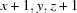; (vi) 
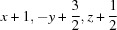
; (vii) 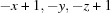; (viii) 
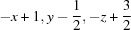
.

**Fig. 1.**
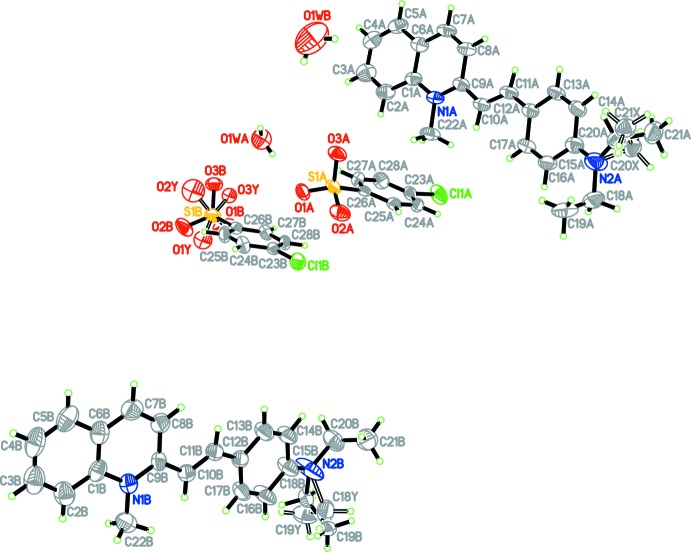
The asymmetric unit of (I) showing 40% probability displacement ellipsoids and the atom-numbering scheme. Open bonds show the minor disorder component.

**Fig. 2.**
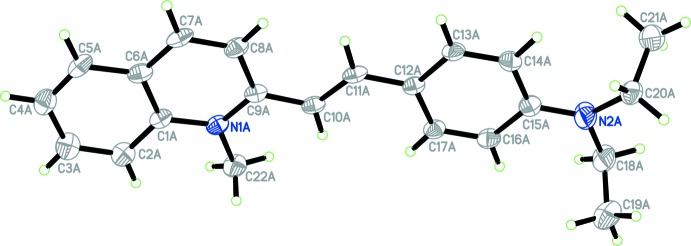
The molecular structure of the major component A showing the configuration of diethylamino group. Only cation A is shown.

**Fig. 3.**
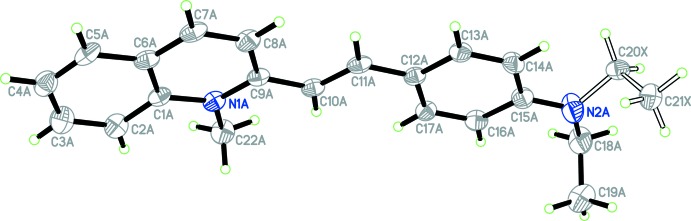
The molecular structure of the minor component X showing the configuration of diethylamino group which differs from that of the major component A. Only cation A is shown.

**Fig. 4.**
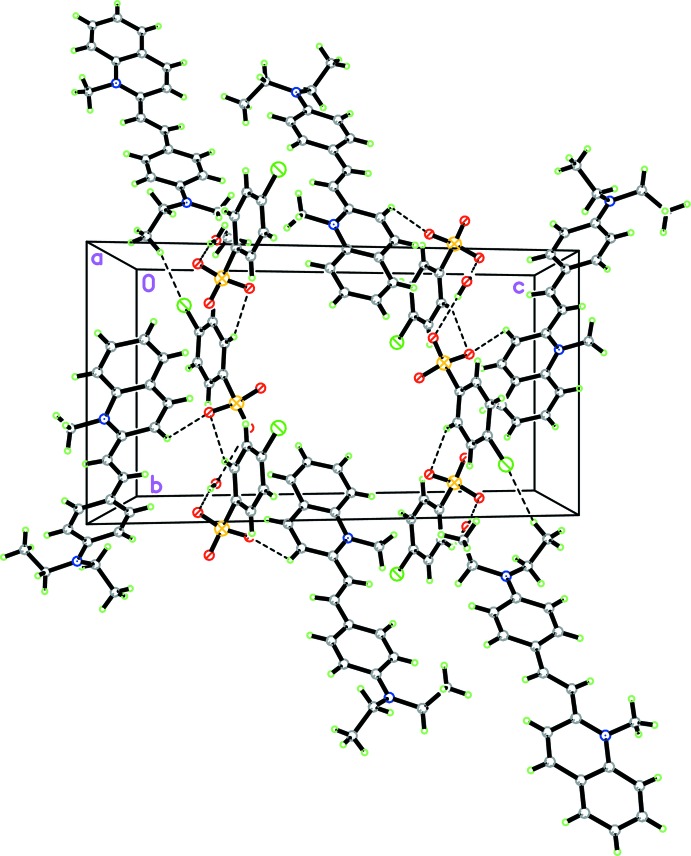
The crystal packing of the major component viewed along the a axis showing chains running along the b axis. The O—H···O hydrogen bonds and weak C—H···O and C—H···Cl interactions are drawn as dashed lines.

**Fig. 5.**
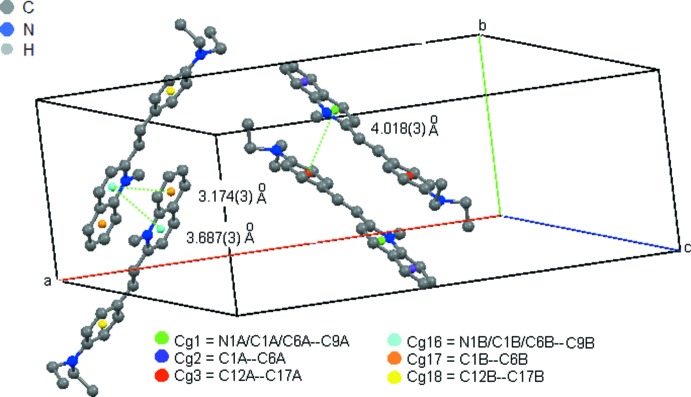
π–π interactions between the aromatic rings of the major disorder components of the cations.

## supplementary crystallographic information

### 1. Comment

The bioactivity of compounds containing the quinolinium chemophore has been the subject of a number of reports (Barchéchath *et al.*, 2005; Chanawanno *et al.*, 2010*a*, 2010*b* and Bolden *et al.*, 2013). The title quinolinium derivative (I) was synthesized and tested for antibacterial activities against gram positive bacteria including *Bacillus subtilis*, *Enterococcus faecalis*, *Staphylococcus aureus*, Methicillin-Resistant *Staphylococcus aureus* and Vancomycin-Resistant *Enterococcus faecalis*, and gram negative bacteria including *Pseudomonas aeruginosa*, *Shigella sonnei* and *Salmonella typhi*. Our antibacterial assay has shown that (I) is strongly active against *B. subtilis* and *P. aeruginosa* with a minimum inhibition concentration (MIC) of 9.37 µg/ml for both strains. In addition (I) also showed moderate activity against *E. faecalis* with an MIC value of 37.5 µg/ml. Herein the crystal structure of (I) is reported.

The asymmetric unit of the title compound (I) (Fig. 1) consists of two C_22_H_25_N_2_^+^ cations, two C_6_H_4_ClO_3_S^-^ anions and two solvent H_2_O molecules [the two molecules are denoted as molecules *A* and *B*]. One ethyl unit of diethylamino group of both cation molecules displays disorder over two positions with refined site occupancy ratios of 0.659 (2):0.341 (2) and 0.501 (2):0.499 (2) for molecules *A* and *B*, respectively. The sulfonate group of the anion *B* also shows disorder over two positions with a refined site occupancy ratio of 0.893 (7):0.107 (7). The cations exist in the *E* configuration with respect to the C10═C11 double bond [1.343 (6) Å] and the torsion angle is C9–C10–C11–C12 of 174.6 (4)° for molecule *A* [the corresponding values are 1.324 (6) Å and -172.5 (4)° for molecule *B*]. The C1–C9/N1 quinolinium ring system is essentially planar with *r.m.s.* deviations of 0.0293 (4) and 0.0198 (5) Å for molecules *A* and *B*, respectively. The dihedral angle between the mean-plane of the quinolinium ring system and that of C12–C17 benzene ring is 10.57 (18) and 14.4 (2) ° for molecules *A* and *B*, respectively. The disorder of the ethyl groups in each cation result in the diethylamino substituents having two different configurations in which the two ethyl groups either point away from one another (Fig. 1 and Fig. 2), or towards one another (Fig. 1 and Fig. 3). The diethylamino substituents also deviate from the planes of the benzene rings to which they are attached as indicated by the torsion angles C15A–N2A–C18A–C19A = -84.1 (7)° and C15A–N2A–C20A–C21A = -96.3 (8)° (major component *A*) and C15A–N2A–C20X–C21X = 100.0 (11)° (minor component *X*). In molecule *B*, the torsion angles C15B–N2B–C20B–C21B = 79.0 (8)° and C15B–N2B–C18B–C19B = -83.7 (10)° (major component *B*) and C15B–N2B–C18Y–C19Y = 112.6 (10)° (minor component *Y*). The bond lengths are in normal ranges (Allen *et al.*, 1987) and comparable to those found in some closely related structures (Chantrapromma *et al.*, 2012; Fun, Kaewmanee *et al.*, 2011, 2013 and Kaewmanee *et al.*, 2010).

In the crystal packing, the cations, anions and water molecules are linked into chains along the [0 1 0] direction by O—H···O_sulfonate_ hydrogen bonds together with weak C—H···O_sulfonate_ and C—H···Cl interactions (Fig. 4 and Table 1). The cations are stacked through π–π interactions with the centroid distances Cg_1_···Cg_1_^iv^ = 3.675 (2) Å, Cg_1_···Cg_2_^iv^ = 4.106 (3) Å, Cg_1_···Cg_3_^ix^ = 4.018 (3) Å, Cg_16_···Cg_16_^x^ = 3.687 (3) Å, Cg_16_···Cg_17_^x^ = 3.714 (3) Å and Cg_16_···Cg_18_^xi^ = 4.162 (3) Å [symmetry codes are as in in Table 1 and (ix) = 1-x, -y, -z; (x) = 2-x, 2-y, 2-z and (xi) = 2-x, 1-y, 2-z]; Cg_1_, Cg_2_, Cg_3_, Cg_16_, Cg_17_ and Cg_18_ are the centroids of the N1A/C1A/C6A–C9A, C1A–C6A, C12A–C17A, N1B/C1B/C6B–C9B, C1B–C6B and C12B–C17B rings, respectively. Fig. 5 shows these π···π interactions only for the major disorder components.

### 2. Experimental

The title compound was prepared by stirring silver (I) 4-chlorobenzenesulfonate (0.95 g, 3.16 mmol) and (*E*)-2-(4-(diethylamino)styryl)-1-methylquinolinium iodide (1.44 g, 3.16 mmol) in methanol (100 ml) for ca. 0.5 h. The precipitate of silver iodide which formed was filtered out and the filtrate was evaporated to give the title compound as a brown solid. Brown block-shaped single crystals of the title compound suitable for X-ray structure determination was recrystallized from ethanol by slow evaporation at room temperature over a few weeks, Mp. 471-473 K.

### 3. Refinement

All H atoms were positioned geometrically and allowed to ride on their parent atoms with d(O-H) = 0.76-0.85 Å, d(C-H) = 0.93 Å for aromatic and CH, 0.97 Å for CH_2_ and 0.96 Å for CH_3_ atoms. The *U*_iso_ values were constrained to be 1.5*U*_eq_ of the carrier atom for water and methyl H atoms and 1.2*U*_eq_ for the remaining H atoms. A rotating group model was used for the methyl groups. The two cations (molecules *A* and *B*) and one anion (molecule *B*) are disordered over two sites with refined site occupancies ratios of 0.659 (2):0.0.341 (2), 0.501 (2):0.499 (2) and 0.893 (7):0.107 (7), respectively.

### Crystal data

**Table:**

C_22_H_25_N_2_^+^·C_6_H_4_ClO_3_S^−^·H_2_O	*F*(000) = 2224
*M**_r_* = 527.07	*D*_x_ = 1.352 Mg m^−^^3^
Monoclinic, *P*2_1_/*c*	Melting point = 471–473 K
Hall symbol: -P 2ybc	Mo *K*α radiation, λ = 0.71073 Å
*a* = 25.814 (4) Å	Cell parameters from 10657 reflections
*b* = 10.5563 (16) Å	θ = 2–26.5°
*c* = 20.333 (3) Å	µ = 0.27 mm^−^^1^
β = 110.883 (2)°	*T* = 100 K
*V* = 5176.8 (14) Å^3^	Block, brown
*Z* = 8	0.31 × 0.19 × 0.15 mm

### Data collection

**Table:**

Bruker SMART APEXII DUO CCD area-detector diffractometer	10657 independent reflections
Radiation source: fine-focus sealed tube	6269 reflections with *I* > 2σ(*I*)
Graphite monochromator	*R*_int_ = 0.064
φ and ω scans	θ_max_ = 26.5°, θ_min_ = 2.0°
Absorption correction: multi-scan (*SADABS*; Bruker, 2009)	*h* = −30→32
*T*_min_ = 0.923, *T*_max_ = 0.961	*k* = −13→13
28821 measured reflections	*l* = −25→25

### Refinement

**Table:**

Refinement on *F*^2^	Primary atom site location: structure-invariant direct methods
Least-squares matrix: full	Secondary atom site location: difference Fourier map
*R*[*F*^2^ > 2σ(*F*^2^)] = 0.076	Hydrogen site location: inferred from neighbouring sites
*wR*(*F*^2^) = 0.201	H-atom parameters constrained
*S* = 1.04	*w* = 1/[σ^2^(*F*_o_^2^) + (0.0652*P*)^2^ + 7.6849*P*] where *P* = (*F*_o_^2^ + 2*F*_c_^2^)/3
10657 reflections	(Δ/σ)_max_ = 0.001
708 parameters	Δρ_max_ = 0.61 e Å^−^^3^
0 restraints	Δρ_min_ = −0.43 e Å^−^^3^

### Special details

**Table:**

Experimental. The crystal was placed in the cold stream of an Oxford Cryosystems Cobra open-flow nitrogen cryostat (Cosier & Glazer, 1986) operating at 100.0 (1) K.
Geometry. All esds (except the esd in the dihedral angle between two l.s. planes) are estimated using the full covariance matrix. The cell esds are taken into account individually in the estimation of esds in distances, angles and torsion angles; correlations between esds in cell parameters are only used when they are defined by crystal symmetry. An approximate (isotropic) treatment of cell esds is used for estimating esds involving l.s. planes.
Refinement. Refinement of F^2^ against ALL reflections. The weighted R-factor wR and goodness of fit S are based on F^2^, conventional R-factors R are based on F, with F set to zero for negative F^2^. The threshold expression of F^2^ > 2sigma(F^2^) is used only for calculating R-factors(gt) etc. and is not relevant to the choice of reflections for refinement. R-factors based on F^2^ are statistically about twice as large as those based on F, and R- factors based on ALL data will be even larger.

### Fractional atomic coordinates and isotropic or equivalent isotropic displacement parameters (Å^2^)

**Table:**

	*x*	*y*	*z*	*U*_iso_*/*U*_eq_	Occ. (<1)
Cl1A	0.27801 (5)	0.17454 (13)	0.13432 (7)	0.0684 (4)	
S1A	0.46207 (4)	0.58226 (11)	0.26409 (5)	0.0415 (3)	
O1A	0.43655 (13)	0.6836 (3)	0.29041 (14)	0.0512 (8)	
O2A	0.50670 (12)	0.5209 (3)	0.31975 (14)	0.0578 (9)	
O3A	0.47678 (12)	0.6192 (3)	0.20454 (14)	0.0538 (8)	
C23A	0.32927 (17)	0.2876 (4)	0.17242 (19)	0.0404 (10)	
C24A	0.38262 (17)	0.2482 (4)	0.2059 (2)	0.0411 (10)	
H24A	0.3915	0.1625	0.2090	0.049*	
C25A	0.42288 (17)	0.3380 (4)	0.23497 (18)	0.0388 (9)	
H25A	0.4594	0.3127	0.2578	0.047*	
C26A	0.40971 (14)	0.4651 (4)	0.23066 (16)	0.0311 (8)	
C27A	0.35560 (17)	0.5029 (4)	0.1966 (2)	0.0415 (10)	
H27A	0.3467	0.5886	0.1933	0.050*	
C28A	0.31469 (17)	0.4142 (5)	0.1674 (2)	0.0483 (11)	
H28A	0.2780	0.4389	0.1447	0.058*	
N1A	0.45312 (13)	0.3584 (3)	0.01622 (15)	0.0406 (8)	
N2A	0.7161 (2)	−0.2368 (5)	0.1056 (2)	0.0799 (15)	
C1A	0.41975 (15)	0.4623 (4)	−0.01354 (19)	0.0402 (10)	
C2A	0.39663 (18)	0.5404 (5)	0.0243 (2)	0.0529 (12)	
H2AA	0.4035	0.5240	0.0716	0.064*	
C3A	0.3643 (2)	0.6400 (5)	−0.0077 (3)	0.0632 (13)	
H3AA	0.3492	0.6909	0.0183	0.076*	
C4A	0.35306 (19)	0.6681 (5)	−0.0783 (3)	0.0570 (12)	
H4AA	0.3298	0.7354	−0.0995	0.068*	
C5A	0.37609 (17)	0.5973 (5)	−0.1160 (2)	0.0520 (12)	
H5AA	0.3695	0.6179	−0.1627	0.062*	
C6A	0.41001 (16)	0.4924 (4)	−0.0853 (2)	0.0443 (10)	
C7A	0.43683 (17)	0.4199 (5)	−0.12117 (19)	0.0489 (11)	
H7AA	0.4310	0.4386	−0.1680	0.059*	
C8A	0.47081 (18)	0.3236 (5)	−0.0897 (2)	0.0489 (11)	
H8AA	0.4885	0.2782	−0.1148	0.059*	
C9A	0.48013 (15)	0.2900 (4)	−0.01798 (18)	0.0388 (9)	
C10A	0.51864 (16)	0.1924 (4)	0.01698 (19)	0.0412 (10)	
H10A	0.5250	0.1775	0.0643	0.049*	
C11A	0.54617 (16)	0.1210 (4)	−0.01445 (19)	0.0435 (10)	
H11A	0.5367	0.1329	−0.0626	0.052*	
C12A	0.58865 (16)	0.0282 (4)	0.01772 (18)	0.0403 (10)	
C13A	0.61718 (17)	−0.0274 (4)	−0.02134 (19)	0.0460 (11)	
H13A	0.6074	−0.0054	−0.0685	0.055*	
C14A	0.65862 (19)	−0.1123 (5)	0.0065 (2)	0.0515 (12)	
H14A	0.6770	−0.1450	−0.0217	0.062*	
C15A	0.67463 (19)	−0.1524 (5)	0.0774 (2)	0.0515 (11)	
C16A	0.64536 (18)	−0.0960 (4)	0.1171 (2)	0.0488 (11)	
H16A	0.6543	−0.1190	0.1640	0.059*	
C17A	0.60460 (16)	−0.0093 (4)	0.08862 (19)	0.0443 (10)	
H17A	0.5868	0.0261	0.1167	0.053*	
C18A	0.7320 (2)	−0.2810 (5)	0.1780 (2)	0.0714 (15)	
H18A	0.7491	−0.3639	0.1819	0.086*	
H18B	0.6990	−0.2901	0.1899	0.086*	
C19A	0.7719 (2)	−0.1921 (6)	0.2300 (2)	0.0733 (16)	
H19A	0.7793	−0.2224	0.2770	0.110*	
H19B	0.7558	−0.1091	0.2251	0.110*	
H19C	0.8059	−0.1882	0.2209	0.110*	
C20A	0.7593 (5)	−0.2587 (10)	0.0703 (5)	0.056 (3)	0.66 (2)
H20A	0.7613	−0.1855	0.0424	0.067*	0.66 (2)
H20B	0.7957	−0.2733	0.1055	0.067*	0.66 (2)
C21A	0.7402 (4)	−0.3732 (10)	0.0241 (7)	0.075 (4)	0.66 (2)
H21A	0.7679	−0.3966	0.0050	0.113*	0.66 (2)
H21B	0.7061	−0.3541	−0.0137	0.113*	0.66 (2)
H21C	0.7343	−0.4422	0.0514	0.113*	0.66 (2)
C20X	0.7305 (7)	−0.3315 (18)	0.0610 (10)	0.049 (6)	0.34 (2)
H20C	0.7008	−0.3424	0.0159	0.059*	0.34 (2)
H20D	0.7401	−0.4128	0.0844	0.059*	0.34 (2)
C21X	0.7817 (9)	−0.265 (2)	0.0534 (10)	0.057 (5)	0.34 (2)
H21D	0.7937	−0.3111	0.0209	0.086*	0.34 (2)
H21E	0.8111	−0.2615	0.0984	0.086*	0.34 (2)
H21F	0.7718	−0.1801	0.0361	0.086*	0.34 (2)
C22A	0.45767 (19)	0.3216 (5)	0.0879 (2)	0.0559 (13)	
H22A	0.4675	0.2336	0.0953	0.084*	
H22B	0.4227	0.3352	0.0936	0.084*	
H22C	0.4857	0.3719	0.1216	0.084*	
Cl1B	0.22680 (5)	0.59222 (12)	0.37098 (6)	0.0612 (3)	
S1B	0.04143 (5)	1.00755 (13)	0.30263 (6)	0.0579 (3)	
O1B	−0.00922 (17)	0.9418 (5)	0.3027 (2)	0.0866 (18)	0.893 (7)
O2B	0.0603 (2)	1.0986 (4)	0.35813 (19)	0.0867 (19)	0.893 (7)
O3B	0.03461 (16)	1.0545 (4)	0.23477 (17)	0.0595 (13)	0.893 (7)
O1Y	0.0265 (16)	1.006 (4)	0.359 (2)	0.075 (11)*	0.107 (7)
O2Y	0.075 (2)	1.122 (5)	0.278 (3)	0.113 (17)*	0.107 (7)
O3Y	0.0062 (12)	0.977 (3)	0.2366 (14)	0.048 (9)*	0.107 (7)
C23B	0.17478 (17)	0.7065 (4)	0.35240 (19)	0.0402 (10)	
C24B	0.18873 (19)	0.8286 (5)	0.3746 (2)	0.0515 (11)	
H24B	0.2254	0.8500	0.3997	0.062*	
C25B	0.14773 (19)	0.9191 (4)	0.3592 (2)	0.0501 (11)	
H25B	0.1570	1.0021	0.3741	0.060*	
C26B	0.09301 (17)	0.8887 (4)	0.32205 (19)	0.0409 (10)	
C27B	0.08004 (18)	0.7646 (4)	0.3010 (2)	0.0474 (11)	
H27B	0.0433	0.7425	0.2763	0.057*	
C28B	0.12075 (18)	0.6726 (4)	0.3161 (2)	0.0449 (10)	
H28B	0.1117	0.5892	0.3018	0.054*	
N1B	1.03757 (14)	0.8261 (4)	1.05282 (18)	0.0477 (9)	
N2B	0.7638 (2)	0.2637 (6)	0.8629 (2)	0.100 (2)	
C1B	1.07121 (16)	0.9334 (4)	1.0564 (2)	0.0478 (11)	
C2B	1.0951 (2)	1.0019 (5)	1.1184 (3)	0.0644 (14)	
H2BA	1.0896	0.9767	1.1592	0.077*	
C3B	1.1261 (2)	1.1042 (6)	1.1192 (3)	0.0761 (16)	
H3BA	1.1421	1.1485	1.1611	0.091*	
C4B	1.1352 (2)	1.1459 (5)	1.0598 (4)	0.0747 (16)	
H4BA	1.1571	1.2170	1.0623	0.090*	
C5B	1.1124 (2)	1.0837 (5)	0.9981 (3)	0.0654 (14)	
H5BA	1.1185	1.1117	0.9581	0.079*	
C6B	1.07887 (18)	0.9748 (5)	0.9949 (3)	0.0537 (12)	
C7B	1.0522 (2)	0.9091 (5)	0.9319 (3)	0.0593 (13)	
H7BA	1.0571	0.9355	0.8909	0.071*	
C8B	1.01959 (19)	0.8085 (4)	0.9299 (2)	0.0533 (12)	
H8BA	1.0021	0.7671	0.8873	0.064*	
C9B	1.01126 (17)	0.7638 (4)	0.9921 (2)	0.0426 (10)	
C10B	0.97260 (17)	0.6636 (4)	0.9875 (2)	0.0447 (10)	
H10B	0.9677	0.6381	1.0287	0.054*	
C11B	0.94348 (18)	0.6051 (4)	0.9283 (2)	0.0486 (11)	
H11B	0.9526	0.6256	0.8893	0.058*	
C12B	0.89936 (17)	0.5137 (4)	0.9161 (2)	0.0441 (10)	
C13B	0.8711 (2)	0.4738 (5)	0.8474 (2)	0.0578 (13)	
H13B	0.8823	0.5046	0.8118	0.069*	
C14B	0.82772 (19)	0.3919 (5)	0.8300 (2)	0.0562 (13)	
H14B	0.8104	0.3683	0.7832	0.067*	
C15B	0.8085 (2)	0.3420 (5)	0.8811 (2)	0.0624 (14)	
C16B	0.8378 (2)	0.3800 (6)	0.9517 (2)	0.0713 (16)	
H16B	0.8274	0.3478	0.9878	0.086*	
C17B	0.88144 (19)	0.4641 (5)	0.9675 (2)	0.0527 (12)	
H17B	0.8994	0.4884	1.0141	0.063*	
C18B	0.7377 (4)	0.2303 (11)	0.9135 (6)	0.039 (4)	0.50 (2)
H18C	0.6995	0.2045	0.8896	0.047*	0.50 (2)
H18D	0.7385	0.3013	0.9441	0.047*	0.50 (2)
C19B	0.7729 (7)	0.1191 (16)	0.9558 (7)	0.059 (4)	0.50 (2)
H19D	0.7571	0.0886	0.9890	0.089*	0.50 (2)
H19E	0.8101	0.1476	0.9806	0.089*	0.50 (2)
H19F	0.7734	0.0519	0.9242	0.089*	0.50 (2)
C18Y	0.7654 (8)	0.1500 (17)	0.9252 (11)	0.073 (5)	0.50 (2)
H18E	0.7575	0.0654	0.9055	0.087*	0.50 (2)
H18F	0.7998	0.1505	0.9655	0.087*	0.50 (2)
C19Y	0.7197 (6)	0.2047 (14)	0.9404 (7)	0.081 (5)	0.50 (2)
H19G	0.7132	0.1565	0.9768	0.122*	0.50 (2)
H19H	0.6870	0.2033	0.8987	0.122*	0.50 (2)
H19I	0.7284	0.2906	0.9558	0.122*	0.50 (2)
C20B	0.7343 (2)	0.2220 (6)	0.7893 (2)	0.0650 (14)	
H20E	0.7304	0.2936	0.7580	0.078*	
H20F	0.6974	0.1936	0.7845	0.078*	
C21B	0.7635 (2)	0.1176 (6)	0.7675 (3)	0.0732 (15)	
H21G	0.7438	0.0970	0.7190	0.110*	
H21H	0.7652	0.0443	0.7961	0.110*	
H21I	0.8005	0.1442	0.7733	0.110*	
C22B	1.0325 (2)	0.7835 (5)	1.1179 (2)	0.0666 (14)	
H22D	1.0187	0.6982	1.1123	0.100*	
H22E	1.0071	0.8377	1.1296	0.100*	
H22F	1.0681	0.7865	1.1550	0.100*	
O1WA	0.40302 (13)	0.9141 (3)	0.21471 (18)	0.0667 (9)	
H1WB	0.9111	0.8460	0.2208	0.100*	
H2WB	0.9173	0.7813	0.1665	0.100*	
O1WB	0.9093 (2)	0.8517 (5)	0.1784 (3)	0.1310 (19)	
H1WA	0.4309	0.9315	0.2043	0.197*	
H2WA	0.4077	0.8524	0.2351	0.197*	

### Atomic displacement parameters (Å^2^)

**Table:**

	*U*^11^	*U*^22^	*U*^33^	*U*^12^	*U*^13^	*U*^23^
Cl1A	0.0795 (8)	0.0702 (9)	0.0648 (7)	−0.0410 (7)	0.0372 (6)	−0.0300 (7)
S1A	0.0496 (6)	0.0487 (7)	0.0280 (4)	−0.0144 (5)	0.0162 (4)	−0.0073 (4)
O1A	0.071 (2)	0.0453 (18)	0.0434 (15)	−0.0149 (16)	0.0283 (15)	−0.0155 (14)
O2A	0.0490 (17)	0.079 (2)	0.0356 (14)	−0.0096 (17)	0.0026 (13)	−0.0031 (15)
O3A	0.0645 (19)	0.065 (2)	0.0391 (15)	−0.0277 (17)	0.0270 (14)	−0.0083 (14)
C23A	0.053 (2)	0.038 (2)	0.0353 (19)	−0.017 (2)	0.0216 (18)	−0.0094 (18)
C24A	0.060 (3)	0.030 (2)	0.044 (2)	0.000 (2)	0.032 (2)	0.0002 (19)
C25A	0.047 (2)	0.043 (3)	0.0308 (18)	0.004 (2)	0.0203 (17)	0.0016 (18)
C26A	0.038 (2)	0.036 (2)	0.0213 (15)	−0.0057 (18)	0.0139 (14)	−0.0020 (15)
C27A	0.052 (2)	0.032 (2)	0.040 (2)	0.005 (2)	0.0160 (19)	0.0017 (18)
C28A	0.040 (2)	0.055 (3)	0.046 (2)	−0.002 (2)	0.0107 (19)	−0.006 (2)
N1A	0.0411 (18)	0.054 (2)	0.0299 (15)	−0.0039 (18)	0.0160 (14)	0.0051 (16)
N2A	0.099 (3)	0.095 (4)	0.047 (2)	0.049 (3)	0.027 (2)	0.018 (2)
C1A	0.035 (2)	0.048 (3)	0.0354 (19)	−0.010 (2)	0.0100 (16)	0.0043 (19)
C2A	0.050 (3)	0.064 (3)	0.051 (2)	−0.001 (3)	0.026 (2)	0.006 (2)
C3A	0.056 (3)	0.070 (4)	0.070 (3)	0.003 (3)	0.030 (3)	0.007 (3)
C4A	0.049 (3)	0.054 (3)	0.064 (3)	−0.002 (2)	0.014 (2)	0.011 (3)
C5A	0.039 (2)	0.063 (3)	0.043 (2)	−0.008 (2)	0.0005 (19)	0.014 (2)
C6A	0.036 (2)	0.058 (3)	0.037 (2)	−0.014 (2)	0.0114 (17)	−0.002 (2)
C7A	0.048 (2)	0.068 (3)	0.0252 (18)	−0.011 (2)	0.0063 (17)	0.008 (2)
C8A	0.052 (2)	0.063 (3)	0.0303 (19)	0.001 (2)	0.0139 (18)	0.007 (2)
C9A	0.034 (2)	0.051 (3)	0.0316 (18)	−0.010 (2)	0.0125 (16)	−0.0017 (18)
C10A	0.044 (2)	0.053 (3)	0.0271 (18)	−0.008 (2)	0.0129 (17)	0.0007 (18)
C11A	0.044 (2)	0.059 (3)	0.0250 (17)	−0.012 (2)	0.0087 (16)	0.0010 (18)
C12A	0.041 (2)	0.048 (3)	0.0304 (18)	−0.004 (2)	0.0112 (16)	0.0003 (18)
C13A	0.051 (2)	0.059 (3)	0.0266 (18)	0.003 (2)	0.0123 (17)	0.0004 (19)
C14A	0.062 (3)	0.060 (3)	0.034 (2)	0.011 (3)	0.019 (2)	−0.001 (2)
C15A	0.057 (3)	0.056 (3)	0.039 (2)	0.006 (2)	0.013 (2)	0.003 (2)
C16A	0.058 (3)	0.057 (3)	0.0323 (19)	0.000 (2)	0.0169 (19)	0.006 (2)
C17A	0.044 (2)	0.059 (3)	0.0317 (19)	0.000 (2)	0.0153 (17)	0.001 (2)
C18A	0.087 (4)	0.068 (4)	0.055 (3)	0.025 (3)	0.020 (3)	0.024 (3)
C19A	0.077 (3)	0.093 (5)	0.048 (3)	0.008 (3)	0.021 (3)	0.033 (3)
C20A	0.061 (7)	0.045 (5)	0.051 (5)	0.011 (6)	0.008 (4)	0.007 (4)
C21A	0.089 (7)	0.055 (6)	0.069 (7)	0.016 (5)	0.013 (5)	0.000 (5)
C20X	0.054 (9)	0.038 (10)	0.051 (10)	−0.011 (8)	0.014 (7)	0.000 (8)
C21X	0.056 (12)	0.060 (12)	0.060 (10)	0.004 (9)	0.026 (8)	0.009 (8)
C22A	0.066 (3)	0.074 (4)	0.036 (2)	0.007 (3)	0.028 (2)	0.011 (2)
Cl1B	0.0747 (8)	0.0558 (8)	0.0571 (6)	0.0224 (7)	0.0284 (6)	−0.0002 (6)
S1B	0.0768 (8)	0.0564 (8)	0.0458 (6)	0.0258 (7)	0.0285 (6)	0.0085 (6)
O1B	0.069 (3)	0.111 (4)	0.102 (3)	0.033 (3)	0.059 (3)	0.043 (3)
O2B	0.122 (4)	0.082 (3)	0.048 (2)	0.061 (3)	0.020 (2)	−0.009 (2)
O3B	0.077 (3)	0.060 (3)	0.0453 (19)	0.021 (2)	0.0270 (18)	0.0125 (18)
C23B	0.055 (3)	0.038 (2)	0.0343 (19)	0.011 (2)	0.0236 (19)	0.0012 (18)
C24B	0.050 (3)	0.052 (3)	0.053 (2)	−0.004 (2)	0.019 (2)	−0.015 (2)
C25B	0.065 (3)	0.035 (2)	0.057 (3)	−0.004 (2)	0.030 (2)	−0.011 (2)
C26B	0.051 (2)	0.042 (3)	0.0334 (19)	0.003 (2)	0.0192 (18)	0.0005 (18)
C27B	0.051 (2)	0.049 (3)	0.043 (2)	−0.004 (2)	0.0179 (19)	−0.011 (2)
C28B	0.062 (3)	0.033 (2)	0.047 (2)	−0.005 (2)	0.030 (2)	−0.0081 (19)
N1B	0.050 (2)	0.045 (2)	0.049 (2)	0.0006 (19)	0.0180 (16)	−0.0041 (17)
N2B	0.111 (4)	0.154 (5)	0.045 (2)	−0.086 (4)	0.039 (2)	−0.033 (3)
C1B	0.037 (2)	0.037 (3)	0.067 (3)	0.003 (2)	0.015 (2)	0.003 (2)
C2B	0.052 (3)	0.064 (4)	0.066 (3)	0.000 (3)	0.007 (2)	−0.010 (3)
C3B	0.055 (3)	0.062 (4)	0.094 (4)	−0.006 (3)	0.006 (3)	−0.013 (3)
C4B	0.049 (3)	0.048 (3)	0.118 (5)	−0.005 (3)	0.018 (3)	−0.008 (4)
C5B	0.054 (3)	0.049 (3)	0.101 (4)	0.000 (3)	0.035 (3)	0.008 (3)
C6B	0.043 (2)	0.046 (3)	0.071 (3)	0.010 (2)	0.020 (2)	−0.005 (2)
C7B	0.067 (3)	0.058 (3)	0.065 (3)	0.003 (3)	0.038 (3)	0.006 (3)
C8B	0.063 (3)	0.047 (3)	0.057 (3)	−0.011 (2)	0.030 (2)	0.000 (2)
C9B	0.050 (2)	0.035 (2)	0.044 (2)	0.004 (2)	0.0179 (19)	−0.0065 (19)
C10B	0.052 (2)	0.042 (3)	0.043 (2)	−0.003 (2)	0.0211 (19)	0.000 (2)
C11B	0.064 (3)	0.046 (3)	0.045 (2)	−0.005 (2)	0.030 (2)	−0.002 (2)
C12B	0.055 (2)	0.039 (3)	0.045 (2)	−0.003 (2)	0.0257 (19)	−0.0014 (19)
C13B	0.073 (3)	0.063 (3)	0.044 (2)	−0.019 (3)	0.029 (2)	−0.010 (2)
C14B	0.064 (3)	0.070 (4)	0.039 (2)	−0.013 (3)	0.025 (2)	−0.009 (2)
C15B	0.066 (3)	0.084 (4)	0.042 (2)	−0.030 (3)	0.026 (2)	−0.014 (2)
C16B	0.088 (4)	0.096 (4)	0.040 (2)	−0.040 (3)	0.036 (2)	−0.018 (3)
C17B	0.063 (3)	0.057 (3)	0.040 (2)	−0.011 (3)	0.021 (2)	−0.013 (2)
C18B	0.035 (5)	0.045 (7)	0.048 (6)	−0.016 (5)	0.027 (4)	−0.013 (5)
C19B	0.091 (9)	0.045 (9)	0.036 (6)	−0.021 (7)	0.016 (7)	0.003 (5)
C18Y	0.101 (12)	0.045 (10)	0.081 (13)	−0.007 (9)	0.043 (11)	−0.007 (9)
C19Y	0.082 (10)	0.108 (11)	0.061 (7)	−0.012 (8)	0.035 (7)	0.011 (7)
C20B	0.056 (3)	0.085 (4)	0.053 (3)	−0.021 (3)	0.019 (2)	−0.020 (3)
C21B	0.072 (3)	0.073 (4)	0.068 (3)	−0.008 (3)	0.017 (3)	−0.003 (3)
C22B	0.081 (3)	0.069 (4)	0.048 (3)	−0.011 (3)	0.020 (2)	−0.003 (2)
O1WA	0.0540 (19)	0.072 (2)	0.074 (2)	0.0148 (18)	0.0227 (17)	0.0020 (19)
O1WB	0.103 (4)	0.110 (4)	0.169 (5)	0.040 (3)	0.036 (3)	0.045 (4)

### Geometric parameters (Å, º)

**Table:**

Cl1A—C23A	1.745 (4)	S1B—O2B	1.430 (4)
S1A—O3A	1.446 (3)	S1B—O1B	1.481 (4)
S1A—O2A	1.449 (3)	S1B—O2Y	1.67 (5)
S1A—O1A	1.455 (3)	S1B—C26B	1.768 (4)
S1A—C26A	1.779 (4)	C23B—C24B	1.371 (6)
C23A—C24A	1.366 (6)	C23B—C28B	1.373 (6)
C23A—C28A	1.382 (6)	C24B—C25B	1.377 (6)
C24A—C25A	1.375 (6)	C24B—H24B	0.9300
C24A—H24A	0.9300	C25B—C26B	1.382 (6)
C25A—C26A	1.379 (5)	C25B—H25B	0.9300
C25A—H25A	0.9300	C26B—C27B	1.382 (6)
C26A—C27A	1.378 (5)	C27B—C28B	1.383 (6)
C27A—C28A	1.378 (6)	C27B—H27B	0.9300
C27A—H27A	0.9300	C28B—H28B	0.9300
C28A—H28A	0.9300	N1B—C9B	1.349 (5)
N1A—C9A	1.355 (5)	N1B—C1B	1.413 (6)
N1A—C1A	1.393 (5)	N1B—C22B	1.448 (5)
N1A—C22A	1.473 (5)	N2B—C15B	1.359 (6)
N2A—C15A	1.352 (6)	N2B—C18B	1.460 (13)
N2A—C18A	1.457 (6)	N2B—C20B	1.483 (6)
N2A—C20X	1.48 (2)	N2B—C18Y	1.74 (2)
N2A—C20A	1.546 (15)	C1B—C2B	1.392 (6)
C1A—C2A	1.398 (6)	C1B—C6B	1.404 (6)
C1A—C6A	1.425 (5)	C2B—C3B	1.341 (7)
C2A—C3A	1.356 (7)	C2B—H2BA	0.9300
C2A—H2AA	0.9300	C3B—C4B	1.381 (8)
C3A—C4A	1.392 (6)	C3B—H3BA	0.9300
C3A—H3AA	0.9300	C4B—C5B	1.351 (8)
C4A—C5A	1.350 (6)	C4B—H4BA	0.9300
C4A—H4AA	0.9300	C5B—C6B	1.426 (7)
C5A—C6A	1.411 (6)	C5B—H5BA	0.9300
C5A—H5AA	0.9300	C6B—C7B	1.402 (7)
C6A—C7A	1.400 (6)	C7B—C8B	1.346 (6)
C7A—C8A	1.346 (6)	C7B—H7BA	0.9300
C7A—H7AA	0.9300	C8B—C9B	1.438 (6)
C8A—C9A	1.435 (5)	C8B—H8BA	0.9300
C8A—H8AA	0.9300	C9B—C10B	1.434 (6)
C9A—C10A	1.433 (6)	C10B—C11B	1.324 (6)
C10A—C11A	1.343 (6)	C10B—H10B	0.9300
C10A—H10A	0.9300	C11B—C12B	1.445 (6)
C11A—C12A	1.442 (6)	C11B—H11B	0.9300
C11A—H11A	0.9300	C12B—C17B	1.387 (5)
C12A—C13A	1.391 (5)	C12B—C13B	1.392 (6)
C12A—C17A	1.408 (5)	C13B—C14B	1.357 (6)
C13A—C14A	1.355 (6)	C13B—H13B	0.9300
C13A—H13A	0.9300	C14B—C15B	1.403 (6)
C14A—C15A	1.416 (6)	C14B—H14B	0.9300
C14A—H14A	0.9300	C15B—C16B	1.422 (6)
C15A—C16A	1.418 (6)	C16B—C17B	1.379 (6)
C16A—C17A	1.358 (6)	C16B—H16B	0.9300
C16A—H16A	0.9300	C17B—H17B	0.9300
C17A—H17A	0.9300	C18B—C19B	1.55 (2)
C18A—C19A	1.511 (8)	C18B—H18C	0.9700
C18A—H18A	0.9700	C18B—H18D	0.9700
C18A—H18B	0.9700	C19B—H19D	0.9600
C19A—H19A	0.9600	C19B—H19E	0.9600
C19A—H19B	0.9600	C19B—H19F	0.9600
C19A—H19C	0.9600	C18Y—C19Y	1.44 (3)
C20A—C21A	1.503 (17)	C18Y—H18E	0.9700
C20A—H20A	0.9700	C18Y—H18F	0.9700
C20A—H20B	0.9700	C19Y—H19G	0.9600
C21A—H21A	0.9600	C19Y—H19H	0.9600
C21A—H21B	0.9600	C19Y—H19I	0.9600
C21A—H21C	0.9600	C20B—C21B	1.490 (7)
C20X—C21X	1.55 (3)	C20B—H20E	0.9700
C20X—H20C	0.9700	C20B—H20F	0.9700
C20X—H20D	0.9700	C21B—H21G	0.9600
C21X—H21D	0.9600	C21B—H21H	0.9600
C21X—H21E	0.9600	C21B—H21I	0.9600
C21X—H21F	0.9600	C22B—H22D	0.9600
C22A—H22A	0.9600	C22B—H22E	0.9600
C22A—H22B	0.9600	C22B—H22F	0.9600
C22A—H22C	0.9600	O1WA—H1WA	0.8388
Cl1B—C23B	1.743 (4)	O1WA—H2WA	0.7583
S1B—O1Y	1.34 (4)	O1WB—H1WB	0.8496
S1B—O3Y	1.36 (3)	O1WB—H2WB	0.8297
S1B—O3B	1.417 (3)		
			
O3A—S1A—O2A	113.70 (18)	O2B—S1B—O1B	111.5 (3)
O3A—S1A—O1A	113.45 (19)	O1Y—S1B—O2Y	127 (2)
O2A—S1A—O1A	112.33 (18)	O3Y—S1B—O2Y	96 (2)
O3A—S1A—C26A	104.97 (16)	O3B—S1B—O2Y	49.6 (17)
O2A—S1A—C26A	105.79 (19)	O2B—S1B—O2Y	71.5 (17)
O1A—S1A—C26A	105.65 (17)	O1B—S1B—O2Y	153.8 (17)
C24A—C23A—C28A	122.1 (4)	O1Y—S1B—C26B	103.0 (16)
C24A—C23A—Cl1A	118.9 (3)	O3Y—S1B—C26B	103.8 (12)
C28A—C23A—Cl1A	119.0 (3)	O3B—S1B—C26B	106.73 (19)
C23A—C24A—C25A	118.6 (4)	O2B—S1B—C26B	106.4 (2)
C23A—C24A—H24A	120.7	O1B—S1B—C26B	105.2 (2)
C25A—C24A—H24A	120.7	O2Y—S1B—C26B	98.3 (16)
C24A—C25A—C26A	120.8 (4)	C24B—C23B—C28B	121.3 (4)
C24A—C25A—H25A	119.6	C24B—C23B—Cl1B	119.2 (3)
C26A—C25A—H25A	119.6	C28B—C23B—Cl1B	119.5 (3)
C27A—C26A—C25A	119.7 (4)	C23B—C24B—C25B	119.2 (4)
C27A—C26A—S1A	119.1 (3)	C23B—C24B—H24B	120.4
C25A—C26A—S1A	121.0 (3)	C25B—C24B—H24B	120.4
C28A—C27A—C26A	120.2 (4)	C24B—C25B—C26B	121.1 (4)
C28A—C27A—H27A	119.9	C24B—C25B—H25B	119.5
C26A—C27A—H27A	119.9	C26B—C25B—H25B	119.5
C27A—C28A—C23A	118.6 (4)	C25B—C26B—C27B	118.5 (4)
C27A—C28A—H28A	120.7	C25B—C26B—S1B	119.9 (3)
C23A—C28A—H28A	120.7	C27B—C26B—S1B	121.6 (3)
C9A—N1A—C1A	123.2 (3)	C26B—C27B—C28B	121.1 (4)
C9A—N1A—C22A	119.6 (4)	C26B—C27B—H27B	119.5
C1A—N1A—C22A	117.2 (3)	C28B—C27B—H27B	119.5
C15A—N2A—C18A	122.4 (4)	C23B—C28B—C27B	118.8 (4)
C15A—N2A—C20X	121.1 (7)	C23B—C28B—H28B	120.6
C18A—N2A—C20X	111.1 (7)	C27B—C28B—H28B	120.6
C15A—N2A—C20A	119.6 (5)	C9B—N1B—C1B	122.5 (4)
C18A—N2A—C20A	115.6 (5)	C9B—N1B—C22B	120.3 (4)
N1A—C1A—C2A	123.0 (3)	C1B—N1B—C22B	117.2 (4)
N1A—C1A—C6A	118.5 (4)	C15B—N2B—C18B	121.0 (5)
C2A—C1A—C6A	118.5 (4)	C15B—N2B—C20B	122.5 (4)
C3A—C2A—C1A	120.3 (4)	C18B—N2B—C20B	116.0 (5)
C3A—C2A—H2AA	119.9	C15B—N2B—C18Y	115.2 (7)
C1A—C2A—H2AA	119.9	C20B—N2B—C18Y	113.6 (7)
C2A—C3A—C4A	121.7 (5)	C2B—C1B—C6B	119.1 (5)
C2A—C3A—H3AA	119.2	C2B—C1B—N1B	121.9 (4)
C4A—C3A—H3AA	119.2	C6B—C1B—N1B	118.9 (4)
C5A—C4A—C3A	119.7 (5)	C3B—C2B—C1B	120.0 (5)
C5A—C4A—H4AA	120.2	C3B—C2B—H2BA	120.0
C3A—C4A—H4AA	120.2	C1B—C2B—H2BA	120.0
C4A—C5A—C6A	120.9 (4)	C2B—C3B—C4B	122.2 (6)
C4A—C5A—H5AA	119.6	C2B—C3B—H3BA	118.9
C6A—C5A—H5AA	119.6	C4B—C3B—H3BA	118.9
C7A—C6A—C5A	122.9 (4)	C5B—C4B—C3B	120.2 (5)
C7A—C6A—C1A	118.2 (4)	C5B—C4B—H4BA	119.9
C5A—C6A—C1A	118.9 (4)	C3B—C4B—H4BA	119.9
C8A—C7A—C6A	121.7 (4)	C4B—C5B—C6B	119.5 (5)
C8A—C7A—H7AA	119.2	C4B—C5B—H5BA	120.3
C6A—C7A—H7AA	119.2	C6B—C5B—H5BA	120.3
C7A—C8A—C9A	121.0 (4)	C7B—C6B—C1B	118.7 (4)
C7A—C8A—H8AA	119.5	C7B—C6B—C5B	122.2 (5)
C9A—C8A—H8AA	119.5	C1B—C6B—C5B	119.0 (5)
N1A—C9A—C10A	121.4 (3)	C8B—C7B—C6B	121.1 (4)
N1A—C9A—C8A	117.3 (4)	C8B—C7B—H7BA	119.5
C10A—C9A—C8A	121.2 (4)	C6B—C7B—H7BA	119.5
C11A—C10A—C9A	124.0 (3)	C7B—C8B—C9B	121.3 (4)
C11A—C10A—H10A	118.0	C7B—C8B—H8BA	119.3
C9A—C10A—H10A	118.0	C9B—C8B—H8BA	119.3
C10A—C11A—C12A	127.7 (3)	N1B—C9B—C10B	122.1 (4)
C10A—C11A—H11A	116.2	N1B—C9B—C8B	117.4 (4)
C12A—C11A—H11A	116.2	C10B—C9B—C8B	120.3 (4)
C13A—C12A—C17A	116.4 (4)	C11B—C10B—C9B	124.0 (4)
C13A—C12A—C11A	120.0 (3)	C11B—C10B—H10B	118.0
C17A—C12A—C11A	123.6 (4)	C9B—C10B—H10B	118.0
C14A—C13A—C12A	122.7 (4)	C10B—C11B—C12B	128.6 (4)
C14A—C13A—H13A	118.6	C10B—C11B—H11B	115.7
C12A—C13A—H13A	118.6	C12B—C11B—H11B	115.7
C13A—C14A—C15A	121.6 (4)	C17B—C12B—C13B	116.5 (4)
C13A—C14A—H14A	119.2	C17B—C12B—C11B	125.3 (4)
C15A—C14A—H14A	119.2	C13B—C12B—C11B	118.2 (4)
N2A—C15A—C14A	121.9 (4)	C14B—C13B—C12B	122.9 (4)
N2A—C15A—C16A	122.5 (4)	C14B—C13B—H13B	118.6
C14A—C15A—C16A	115.6 (4)	C12B—C13B—H13B	118.6
C17A—C16A—C15A	122.0 (4)	C13B—C14B—C15B	121.5 (4)
C17A—C16A—H16A	119.0	C13B—C14B—H14B	119.2
C15A—C16A—H16A	119.0	C15B—C14B—H14B	119.2
C16A—C17A—C12A	121.7 (4)	N2B—C15B—C14B	121.1 (4)
C16A—C17A—H17A	119.2	N2B—C15B—C16B	122.8 (4)
C12A—C17A—H17A	119.2	C14B—C15B—C16B	116.1 (4)
N2A—C18A—C19A	112.6 (5)	C17B—C16B—C15B	120.9 (4)
N2A—C18A—H18A	109.1	C17B—C16B—H16B	119.6
C19A—C18A—H18A	109.1	C15B—C16B—H16B	119.6
N2A—C18A—H18B	109.1	C16B—C17B—C12B	122.1 (4)
C19A—C18A—H18B	109.1	C16B—C17B—H17B	118.9
H18A—C18A—H18B	107.8	C12B—C17B—H17B	118.9
C18A—C19A—H19A	109.5	N2B—C18B—C19B	104.4 (10)
C18A—C19A—H19B	109.5	N2B—C18B—H18C	110.9
H19A—C19A—H19B	109.5	C19B—C18B—H18C	110.9
C18A—C19A—H19C	109.5	N2B—C18B—H18D	110.9
H19A—C19A—H19C	109.5	C19B—C18B—H18D	110.9
H19B—C19A—H19C	109.5	H18C—C18B—H18D	108.9
C21A—C20A—N2A	106.3 (12)	C19Y—C18Y—N2B	94.1 (15)
C21A—C20A—H20A	110.5	C19Y—C18Y—H18E	112.9
N2A—C20A—H20A	110.5	N2B—C18Y—H18E	112.9
C21A—C20A—H20B	110.5	C19Y—C18Y—H18F	112.9
N2A—C20A—H20B	110.5	N2B—C18Y—H18F	112.9
H20A—C20A—H20B	108.7	H18E—C18Y—H18F	110.3
N2A—C20X—C21X	99.4 (16)	C18Y—C19Y—H19G	109.5
N2A—C20X—H20C	111.9	C18Y—C19Y—H19H	109.5
C21X—C20X—H20C	111.9	H19G—C19Y—H19H	109.5
N2A—C20X—H20D	111.9	C18Y—C19Y—H19I	109.5
C21X—C20X—H20D	111.9	H19G—C19Y—H19I	109.5
H20C—C20X—H20D	109.6	H19H—C19Y—H19I	109.5
C20X—C21X—H21D	109.5	N2B—C20B—C21B	112.8 (5)
C20X—C21X—H21E	109.5	N2B—C20B—H20E	109.0
H21D—C21X—H21E	109.5	C21B—C20B—H20E	109.0
C20X—C21X—H21F	109.5	N2B—C20B—H20F	109.0
H21D—C21X—H21F	109.5	C21B—C20B—H20F	109.0
H21E—C21X—H21F	109.5	H20E—C20B—H20F	107.8
N1A—C22A—H22A	109.5	C20B—C21B—H21G	109.5
N1A—C22A—H22B	109.5	C20B—C21B—H21H	109.5
H22A—C22A—H22B	109.5	H21G—C21B—H21H	109.5
N1A—C22A—H22C	109.5	C20B—C21B—H21I	109.5
H22A—C22A—H22C	109.5	H21G—C21B—H21I	109.5
H22B—C22A—H22C	109.5	H21H—C21B—H21I	109.5
O1Y—S1B—O3Y	123 (2)	N1B—C22B—H22D	109.5
O1Y—S1B—O3B	150.2 (16)	N1B—C22B—H22E	109.5
O3Y—S1B—O3B	46.8 (13)	H22D—C22B—H22E	109.5
O1Y—S1B—O2B	56.5 (18)	N1B—C22B—H22F	109.5
O3Y—S1B—O2B	148.7 (12)	H22D—C22B—H22F	109.5
O3B—S1B—O2B	115.0 (3)	H22E—C22B—H22F	109.5
O1Y—S1B—O1B	58.0 (18)	H1WA—O1WA—H2WA	110.0
O3Y—S1B—O1B	67.2 (13)	H1WB—O1WB—H2WB	107.8
O3B—S1B—O1B	111.2 (3)		
			
C28A—C23A—C24A—C25A	−0.4 (5)	C24B—C25B—C26B—C27B	0.6 (6)
Cl1A—C23A—C24A—C25A	179.1 (3)	C24B—C25B—C26B—S1B	−179.5 (3)
C23A—C24A—C25A—C26A	0.2 (5)	O1Y—S1B—C26B—C25B	−88.0 (19)
C24A—C25A—C26A—C27A	−0.3 (5)	O3Y—S1B—C26B—C25B	142.4 (14)
C24A—C25A—C26A—S1A	−176.7 (3)	O3B—S1B—C26B—C25B	93.9 (4)
O3A—S1A—C26A—C27A	−83.0 (3)	O2B—S1B—C26B—C25B	−29.4 (4)
O2A—S1A—C26A—C27A	156.5 (3)	O1B—S1B—C26B—C25B	−147.9 (4)
O1A—S1A—C26A—C27A	37.2 (3)	O2Y—S1B—C26B—C25B	43.6 (18)
O3A—S1A—C26A—C25A	93.5 (3)	O1Y—S1B—C26B—C27B	91.9 (19)
O2A—S1A—C26A—C25A	−27.0 (3)	O3Y—S1B—C26B—C27B	−37.7 (14)
O1A—S1A—C26A—C25A	−146.3 (3)	O3B—S1B—C26B—C27B	−86.3 (4)
C25A—C26A—C27A—C28A	0.5 (5)	O2B—S1B—C26B—C27B	150.4 (4)
S1A—C26A—C27A—C28A	177.0 (3)	O1B—S1B—C26B—C27B	32.0 (4)
C26A—C27A—C28A—C23A	−0.6 (6)	O2Y—S1B—C26B—C27B	−136.5 (18)
C24A—C23A—C28A—C27A	0.6 (6)	C25B—C26B—C27B—C28B	−0.6 (6)
Cl1A—C23A—C28A—C27A	−178.9 (3)	S1B—C26B—C27B—C28B	179.6 (3)
C9A—N1A—C1A—C2A	−173.2 (4)	C24B—C23B—C28B—C27B	0.9 (6)
C22A—N1A—C1A—C2A	8.1 (6)	Cl1B—C23B—C28B—C27B	−179.3 (3)
C9A—N1A—C1A—C6A	5.0 (6)	C26B—C27B—C28B—C23B	−0.1 (6)
C22A—N1A—C1A—C6A	−173.7 (4)	C9B—N1B—C1B—C2B	175.6 (4)
N1A—C1A—C2A—C3A	−179.7 (4)	C22B—N1B—C1B—C2B	−4.9 (6)
C6A—C1A—C2A—C3A	2.1 (6)	C9B—N1B—C1B—C6B	−1.8 (6)
C1A—C2A—C3A—C4A	−0.1 (7)	C22B—N1B—C1B—C6B	177.7 (4)
C2A—C3A—C4A—C5A	−2.0 (7)	C6B—C1B—C2B—C3B	−1.6 (7)
C3A—C4A—C5A—C6A	2.0 (7)	N1B—C1B—C2B—C3B	−179.1 (4)
C4A—C5A—C6A—C7A	−176.9 (4)	C1B—C2B—C3B—C4B	0.5 (8)
C4A—C5A—C6A—C1A	−0.1 (6)	C2B—C3B—C4B—C5B	0.3 (8)
N1A—C1A—C6A—C7A	−3.2 (6)	C3B—C4B—C5B—C6B	0.0 (8)
C2A—C1A—C6A—C7A	175.0 (4)	C2B—C1B—C6B—C7B	−176.7 (4)
N1A—C1A—C6A—C5A	179.8 (4)	N1B—C1B—C6B—C7B	0.9 (6)
C2A—C1A—C6A—C5A	−1.9 (6)	C2B—C1B—C6B—C5B	1.9 (6)
C5A—C6A—C7A—C8A	177.2 (4)	N1B—C1B—C6B—C5B	179.5 (4)
C1A—C6A—C7A—C8A	0.3 (6)	C4B—C5B—C6B—C7B	177.4 (5)
C6A—C7A—C8A—C9A	1.1 (7)	C4B—C5B—C6B—C1B	−1.1 (7)
C1A—N1A—C9A—C10A	173.3 (4)	C1B—C6B—C7B—C8B	0.3 (7)
C22A—N1A—C9A—C10A	−8.0 (6)	C5B—C6B—C7B—C8B	−178.3 (4)
C1A—N1A—C9A—C8A	−3.6 (6)	C6B—C7B—C8B—C9B	−0.6 (7)
C22A—N1A—C9A—C8A	175.2 (4)	C1B—N1B—C9B—C10B	−173.2 (4)
C7A—C8A—C9A—N1A	0.4 (6)	C22B—N1B—C9B—C10B	7.3 (6)
C7A—C8A—C9A—C10A	−176.5 (4)	C1B—N1B—C9B—C8B	1.5 (6)
N1A—C9A—C10A—C11A	179.4 (4)	C22B—N1B—C9B—C8B	−178.0 (4)
C8A—C9A—C10A—C11A	−3.8 (6)	C7B—C8B—C9B—N1B	−0.3 (7)
C9A—C10A—C11A—C12A	174.6 (4)	C7B—C8B—C9B—C10B	174.5 (4)
C10A—C11A—C12A—C13A	−173.0 (4)	N1B—C9B—C10B—C11B	175.8 (4)
C10A—C11A—C12A—C17A	5.6 (7)	C8B—C9B—C10B—C11B	1.3 (7)
C17A—C12A—C13A—C14A	−0.7 (6)	C9B—C10B—C11B—C12B	−172.5 (4)
C11A—C12A—C13A—C14A	178.0 (4)	C10B—C11B—C12B—C17B	−4.1 (8)
C12A—C13A—C14A—C15A	1.7 (7)	C10B—C11B—C12B—C13B	173.9 (5)
C18A—N2A—C15A—C14A	−178.5 (5)	C17B—C12B—C13B—C14B	0.7 (7)
C20X—N2A—C15A—C14A	−26.7 (11)	C11B—C12B—C13B—C14B	−177.5 (5)
C20A—N2A—C15A—C14A	20.0 (9)	C12B—C13B—C14B—C15B	0.1 (8)
C18A—N2A—C15A—C16A	3.2 (8)	C18B—N2B—C15B—C14B	−168.8 (7)
C20X—N2A—C15A—C16A	154.9 (8)	C20B—N2B—C15B—C14B	2.7 (9)
C20A—N2A—C15A—C16A	−158.4 (6)	C18Y—N2B—C15B—C14B	148.1 (8)
C13A—C14A—C15A—N2A	−179.8 (5)	C18B—N2B—C15B—C16B	10.1 (11)
C13A—C14A—C15A—C16A	−1.3 (7)	C20B—N2B—C15B—C16B	−178.5 (6)
N2A—C15A—C16A—C17A	178.6 (5)	C18Y—N2B—C15B—C16B	−33.1 (10)
C14A—C15A—C16A—C17A	0.1 (7)	C13B—C14B—C15B—N2B	177.6 (6)
C15A—C16A—C17A—C12A	0.8 (7)	C13B—C14B—C15B—C16B	−1.4 (8)
C13A—C12A—C17A—C16A	−0.5 (6)	N2B—C15B—C16B—C17B	−177.1 (6)
C11A—C12A—C17A—C16A	−179.1 (4)	C14B—C15B—C16B—C17B	1.8 (8)
C15A—N2A—C18A—C19A	−84.1 (7)	C15B—C16B—C17B—C12B	−1.1 (8)
C20X—N2A—C18A—C19A	121.6 (8)	C13B—C12B—C17B—C16B	−0.2 (7)
C20A—N2A—C18A—C19A	78.1 (7)	C11B—C12B—C17B—C16B	177.9 (5)
C15A—N2A—C20A—C21A	−96.3 (8)	C15B—N2B—C18B—C19B	−83.7 (10)
C18A—N2A—C20A—C21A	100.9 (7)	C20B—N2B—C18B—C19B	104.4 (8)
C20X—N2A—C20A—C21A	8.0 (10)	C18Y—N2B—C18B—C19B	8.6 (14)
C15A—N2A—C20X—C21X	100.0 (11)	C15B—N2B—C18Y—C19Y	112.6 (10)
C18A—N2A—C20X—C21X	−105.4 (10)	C18B—N2B—C18Y—C19Y	3.8 (8)
C20A—N2A—C20X—C21X	−0.3 (10)	C20B—N2B—C18Y—C19Y	−98.9 (10)
C28B—C23B—C24B—C25B	−0.8 (6)	C15B—N2B—C20B—C21B	79.0 (8)
Cl1B—C23B—C24B—C25B	179.3 (3)	C18B—N2B—C20B—C21B	−109.2 (7)
C23B—C24B—C25B—C26B	0.1 (6)	C18Y—N2B—C20B—C21B	−66.9 (9)

### Hydrogen-bond geometry (Å, º)

**Table:**

*D*—H···*A*	*D*—H	H···*A*	*D*···*A*	*D*—H···*A*
O1*WB*—H1*WB*···O1*B*^i^	0.85	2.36	2.815 (7)	114
O1*WB*—H2*WB*···O2*B*^ii^	0.83	2.12	2.953 (7)	177
O1*WA*—H1*WA*···O2*A*^iii^	0.84	2.07	2.891 (5)	166
O1*WA*—H2*WA*···O1*A*	0.76	2.10	2.844 (4)	169
C8*A*—H8*AA*···O3*A*^iv^	0.93	2.54	3.146 (5)	123
C2*B*—H2*BA*···O3*B*^v^	0.93	2.57	3.314 (7)	137
C11*B*—H11*B*···O1*B*^vi^	0.93	2.41	3.237 (6)	148
C18*Y*—H18*E*···Cl1*A*^vii^	0.97	2.72	3.673 (19)	169
C19*B*—H19*D*···Cl1*B*^viii^	0.96	2.73	3.531 (14)	142
C22*B*—H22*D*···O2*B*^viii^	0.96	2.55	3.259 (7)	131
C25*A*—H25*A*···O3*A*^ii^	0.93	2.56	3.359 (5)	144

Symmetry codes: (i) *x*+1, *y*, *z*; (ii) −*x*+1, *y*−1/2, −*z*+1/2; (iii) −*x*+1, *y*+1/2, −*z*+1/2; (iv) −*x*+1, −*y*+1, −*z*; (v) *x*+1, *y*, *z*+1; (vi) *x*+1, −*y*+3/2, *z*+1/2; (vii) −*x*+1, −*y*, −*z*+1; (viii) −*x*+1, *y*−1/2, −*z*+3/2.
